# Study of the Electronic Structure of Alkali Peroxides and Their Role in the Chemistry of Metal–Oxygen Batteries

**DOI:** 10.1021/acs.jpca.1c07255

**Published:** 2021-10-15

**Authors:** Adriano Pierini, Sergio Brutti, Enrico Bodo

**Affiliations:** † Department of Chemistry, University of Rome “La Sapienza”, P. A. Moro 5, Rome 00185, Italy; ‡ GISEL—Centro di Riferimento Nazionale per i Sistemi di Accumulo Elettrochimico di Energia, INSTM via G. Giusti 9, Firenze 50121, Italy

## Abstract

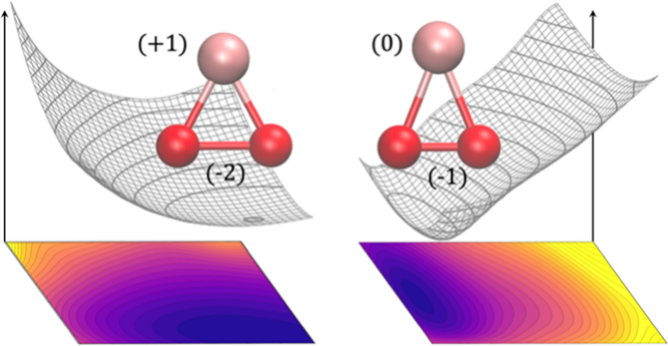

We use a multiconfigurational and correlated ab initio method to investigate the fundamental electronic properties of the peroxide MO_2_^–^ (M = Li and Na) trimer to provide new insights into the rather complex chemistry of aprotic metal–O_2_ batteries. These electrochemical systems are largely based on the electronic properties of superoxide and peroxide of alkali metals. The two compounds differ by stoichiometry: the superoxide is characterized by a M^+^O_2_^–^ formula, while the peroxide is characterized by [M^+^]_2_O_2_^2–^. We show here that both the peroxide and superoxide states necessarily coexist in the MO_2_^–^ trimer and that they correspond to their different electronic states. The energetic prevalence of either one or the other and the range of their coexistence over a subset of the MO_2_^–^ nuclear configurations is calculated and described via a high-level multiconfigurational approach.

## 1 Introduction

Aprotic metal–oxygen batteries^1−3^ (aLOBs) based on the O_2_/Li and possibly on O_2_/Na electrochemistry are a tremendous opportunity and, at the same time, a technological challenge.^4−8^ While their theoretical performance overcomes all other proposed battery chemistries, their practical implementation in real devices is still hampered by several unsolved issues, among which the parasitic release of several degrading byproducts is one of the foremost. In aLOBs based on lithium metal, the electroactive process is a two-step reaction that involves the consecutive reduction of molecular oxygen to superoxide and peroxide anions. This simple reactive pathway is puzzled by the precipitation of both lithium superoxide and lithium peroxide, the chemical disproportionation of lithium superoxide, as well as the concurrent electrochemical/chemical degradation chemistries of the electrolytes and carbonaceous electrodes, leading to the accumulation of byproducts (e.g., lithium carbonate) or gas release (e.g., CO_2_).^1−3^

The chemical disproportionation of metal superoxide in the case of Li ions takes the form
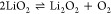
R1and represents a key step in the complex chemistry of Li–air batteries.^9^ In particular, LiO_2_ is a radical intermediate both in the electrochemical reduction of O_2_ and in the oxidation of Li_2_O_2_.^10,11^ Despite its simplicity, reactions such as R1 hide a very complex mechanism that involves singlet–triplet spin intersystem crossing while evolving from two doublet LiO_2_ radicals.^12−14^ Due to the coexistence of electronic states of different multiplicities, the disproportionation product can be either ^1^O_2_ (singlet oxygen) or ^3^O_2_ (triplet, ground-state oxygen).^15,16^ The formation of singlet oxygen, which is a highly reactive biradical molecule, is at the origin of parasitic degradation reactions during electrochemical charge/discharge cycles. As expected, ^1^O_2_ release leads to battery death upon cycling.^16−18^

Despite the huge interest in these systems, many of the underlying chemical processes occurring during the operation of metal–air batteries are still debated, and several computational studies have appeared to try to tackle the fundamental chemical processes that occur.^19^ Generally speaking, the interest in lithium superoxide properties dates back to the 1980s.^20,21^ More recently, calculations of the reactivity of Li superoxide surfaces have been reported by Mo et al.^22^ Other calculations on the bulk phase of Li (su)peroxide have shown the coexistence of superoxide and peroxide ions within the same compound with a Li_3_O_4_ stoichiometry.^23^ Density functional theory (DFT) calculations have been used to characterize the bulk phase of sodium superoxide^24,25^ and have been accompanied with coupled-cluster methods in refs (12, 26) to explore various possible intermediates in the disproportionation reaction of Li and Na superoxides. Very recently, Zaichenko et al. have applied multiconfigurational methods to the study of the dissociation pathways of alkali superoxides.^27^ In a previous work, we have explored the superoxide disproportionation reaction when catalyzed by protons or Li ions using multiconfigurational methods.^14^

From an oversimplified standpoint, the superoxide disproportionation reaction takes place between two O_2_^–^ anions
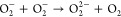
R2where the transfer of a single electron from one of the anions to the other leads to the products (peroxide plus oxygen). Inevitably, the double negatively charged prereaction complex (O_2_®···O_2_®) has little or no chance of forming due to Coulomb repulsion, and, for R2 to occur efficiently, a positively charged catalyst is necessary. A well-known catalytic agent for this reaction is a proton, which partially neutralizes the negative charge and makes the following reaction very efficient.^14,28,29^

In batteries, reaction R2 occurs when two superoxide ions can come into close contact due to the presence of a metallic or protic positively charged center (M^+^ = H^+^, Li^+^, Na^+^, etc.) that allows overcoming the Coulomb repulsion. The reaction, in this case, can be written as
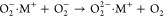
R3Such a process is endergonic for metal cations and exergonic for the proton. In other words, in the absence of protic impurities, it does not appear to be very efficient at moderate temperature and with low overpotentials.

However, a similar reaction with the same reactants can proceed through a different path involving another electronic state, which has lower energy compared to O_2_^2–^·M^+^ and may open the way for a more efficient oxygen release.^30^ This second channel requires the reduction of the metal center, while the superoxide does not change its nature, as shown below in reaction R4
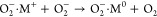
R4In other words, in this channel, molecular oxygen is produced by an electron transfer to the metal. The prevalence of one channel (R3) or the other (R4) is determined by the relative stability of the O_2_^2–^·M^+^ and O_2_^–^·M^0^ species, which simply represent two different electronic states of the same molecule.

This work describes our study of the electronic properties of the MO_2_^–^ species in its singlet multiplicity using multiconfigurational methods, a level required by the diradical nature of its electronic states. Most of the theoretical works cited above have focused on the superoxide side of reaction R3, while, to the best of our knowledge, the possibility of having a concurrent reduction of the metal center (R4) has been, until recently, overlooked. This is probably linked to the fact that MO_2_^–^ is a closed-shell system, and a single-reference calculation typically defaults to the restricted self-consistent field (SCF) solution. The metal reduction channel can only be seen by forcing the SCF to converge to a broken symmetry singlet diradical configuration. As we shall show, it is the broken symmetry solution that often represents the lowest-lying electronic state of MO_2_^–^. Since we are dealing with an open-shell singlet, the system can also be found in the triplet spin arrangement. The existence of the triplet system, however, is not crucial for the following discussion and is addressed in the Supporting Information, Section S4.

It is the purpose of this work to explore the electronic structure of MO_2_^–^ (M = Li, Na) systems using multiconfigurational methods to provide insights into the nature of one of the key molecular partners partaking in the rather complex reactive landscape of (su)peroxide reactions in the presence of alkali ions. Our investigation aims at providing insights into the fundamental chemistry that drives the energy conversion in aLOBs. Despite the apparent simplicity, aLOBs exploit extremely complex chemical processes based on a bunch of concurrent/simultaneous reactions at the solid–liquid interface on heterogeneous electrodes that strongly depend on the surrounding chemical environment (e.g., solvent, salt).^31,32^ A realistic modeling of such systems based on ab initio methods is simply impossible, given the present technical capabilities of high-performance computing. In fact, besides the electrochemical reactions, any reliable model needs to account for the occurrence of parasitic reactions, solvent effects, heterogeneity, precipitation, etc. Thus, given the extreme complexity of the real systems, a reductionist approach is mandatory. In the present work, we step in this direction by focusing on a relatively simple molecular system that, nevertheless, plays a pivotal role in the metal–air battery chemistries and that has never been explored in such detail before. As we shall see, despite its apparent simplicity, it shows a rather surprising complex electronic structure whose features hamper the use of out-of-the-box computational tools based on single-reference ansatz.

## 2 Methods

The energies reported here are based on the CASSCF multiconfigurational methods followed by the evaluations of the correlation energy using a perturbative treatment (MS-CASPT2) on the first three singlet electronic states of MO_2_^–^. The calculations were performed with the OpenMolcas^33^ code package (version 20.10).

The CASSCF wavefunctions were optimized in an active space composed of 10 electrons in seven orbitals. The active space is made of the 2p orbitals of the two oxygen atoms and the 2s or 3s orbital of Li and Na, respectively. Hence, the active space of MO_2_^–^ includes the 3σ, 3σ*, 1π, and 1π* molecular orbitals of O_2_^–^/O_2_^2–^ (see Section S1). A study on the dimension of the active space is presented in the Supporting Information in Section S5.

All calculations, except when specifically noted, have been carried out in the *C*_2*v*_ point group symmetry. This choice has been made to simplify the search for the roots of the MCSCF problem and to help in identifying the correct electronic configurations. Small deviations from the *C*_2*v*_ symmetry do not significantly alter the results, and in any case, the stationary points of the MO_2_^–^ system are naturally characterized by a *C*_2*v*_ geometry.

The correlation was added on top of the 3-state-averaged CASSCF wavefunctions using the Multistate CASPT2^34^ method (MS-CASPT2). The multistate variant turned out to be necessary (instead of plain CASPT2) to correct for the bad description of root-mixing that often emerges (in particular for the *A*_1_ state) at the CASSCF level. A constant imaginary shift of 0.1 a.u. was applied to correct for intruder state singularities along the potential energy surfaces.^35^

**Figure 1:**
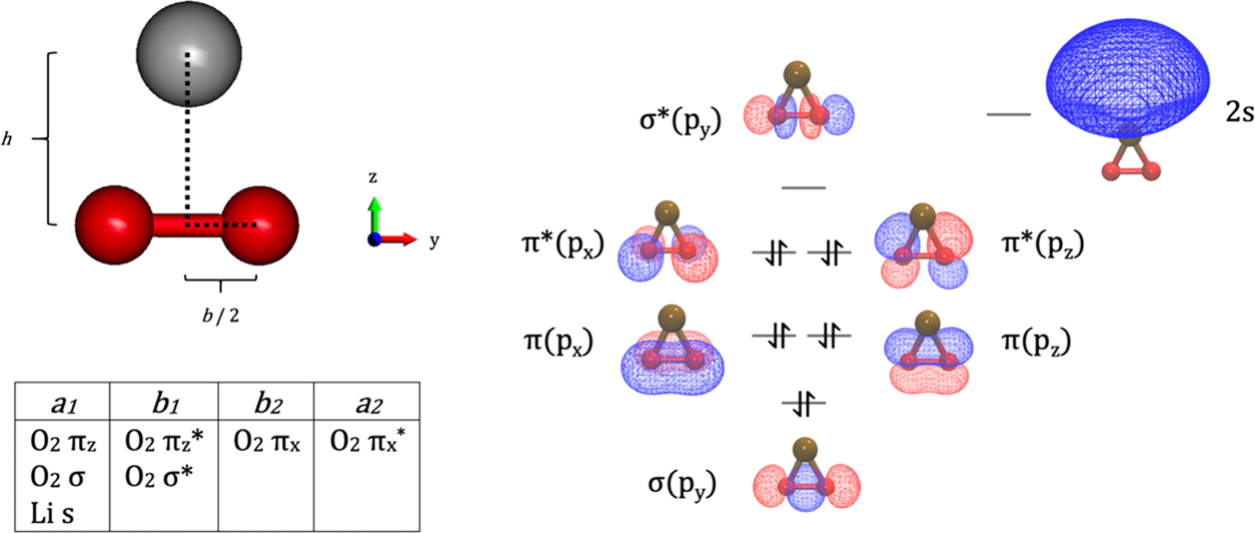
Top left: illustration of the geometric parameters used in the calculations (oxygen atoms are in red and the alkali metal in gray). Bottom left: symmetry properties of the orbitals making the active space. Right: qualitative scheme with the shapes of the active orbitals.

Given the symmetry, the MO_2_^–^ system is an isosceles triangle. The potential energy surfaces (PESs) were generated by varying the O–O distance (the triangle base, *b*) and the distance of the alkali from the geometric center of O–O (the triangle height, *h*) as shown in Figure 1. The geometric grid was produced using increments of 0.05 Å along *b* and *h* starting, respectively, from 1.30 and 1.65 Å (for Li) and from 1.30 and 1.70 (for Na). To plot the results, the raw data have been interpolated on a fine 1000 × 1000 grid using splines. PES calculations were performed using the aug-cc-pVQZ basis set,^36^^36^ while the additional optimizations of the stationary points in each of the PES minimum were done using the aug-cc-pVTZ one and numerical CASPT2 gradients.

## 3 Results and Discussion

The structure of the MO_2_ trimer and the symmetry of the orbitals included in the active space are shown in Figure 1.

The first three electronic states of the trimer have different symmetries and occupations:

- An A_1_ state that has appreciable multideterminantal character. Its leading configuration (typically with weight ∼0.90) is (σ)^2^(π*_z_*)^2^(π*_x_*)^2^(π_z_^*^)^2^(π_x_^*^)^2^(s)^0^(σ*)^0^. Hence, this state corresponds to a filled π* shell and an empty s orbital and represents the O_2_^2–^·M^+^ peroxide state.
- An A_2_ state that is substantially dominated (weight ∼0.99) by single diradical singlet CSF with occupation (σ)^2^(π_z_)^2^(π*_x_*)^2^(π_z_^*^)^2^(π_x_^*^)^1^(s)^1^(σ*)^0^ and corresponds to a superoxide state with a neutral metal O_2_^–^·M^0^.
- Finally, a B_1_ state that is another superoxide largely dominated (weight ∼0.99) by one singlet diradical CSF with the (σ)^2^(π*_z_*)^2^(π*_x_*)^2^(π_z_^*^)^1^(π_x_^*^)^2^(s)^1^(σ*)^0^ configuration.

More details about the electronic configurations can be found in Sections S2 and S3 in the Supporting Information (SI).

Overall, the trimer can assume a peroxide or a superoxide character depending on the relative energies of the above states, whose ordering depends on the geometry of the complex itself. As a rule of thumb, we can anticipate that when the O–O distance is large, the peroxide state A_1_ tends to be the ground state, while for shorter O–O values, the lowest energy state is the superoxide one with symmetry A_2_.

Within the set of geometries sampled here, all of the three electronic states present a minimum energy geometry. The three minima are characterized by different geometries and are reported in Table 1. The absolute energy minima, for both Li and Na, correspond to that of the superoxide A_2_ state, which has a neutral alkali atom (a reduced alkali ion) with an s^1^ electronic configuration. The O–O distance is around 1.4 Å, which is typical of superoxide ions. The O–M distance is 1.8 Å for Li and 2.2 Å for Na. The other superoxide state B_1_ has a minimum located at a similar geometry but with higher energy separated (adiabatically) by ∼0.4 and ∼0.7 eV from the A_2_ minimum for Li and Na, respectively.

**Table 1:**
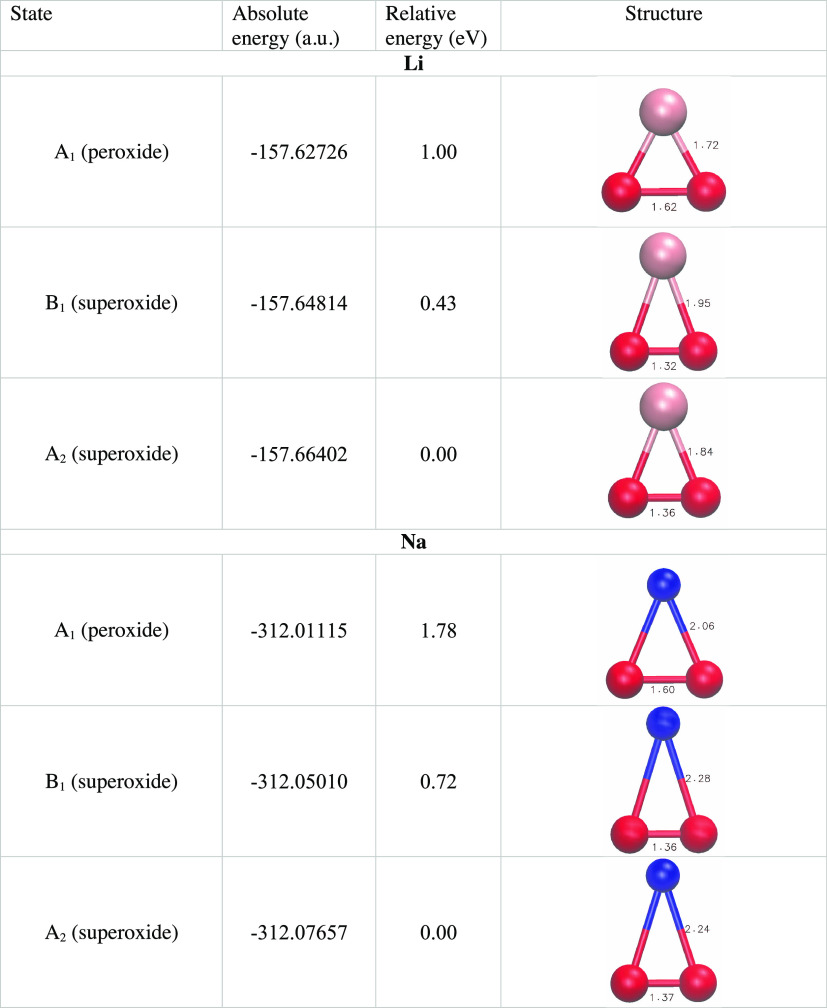
Energies and Structures of the Minimum Points of the Three Electronic States Obtained at the CASPT2/aug-cc-pVTZ Level

The minimum of the peroxide state A_1_ is located 1 and 1.8 eV above the A_2_ one for Li and Na, respectively. This minimum has a geometry with a O–O distance, which is consistent with a peroxide anion (∼1.6 Å) and an M–O distance that shortens with respect to superoxide, and is 1.6 and 2.1 Å for Li and Na, respectively.

As anticipated and quite surprisingly, the ground electronic energy state of the MO_2_^–^ system is not the peroxide but the superoxide with a neutral alkali metal atom. This is, at least, the situation in the gas phase. The tendency for a net energetic preference of the system toward a superoxide configuration is certainly linked to the absence of a solvent medium. Gas phase naturally tends to favor electronic configurations with a smaller degree of charge separation (i.e., the superoxide one). We expect that the energetic differences between the electronic states might be reduced substantially when the system is within a solvent medium that, depending on its polarity and coordination abilities, can stabilize charge-separated configurations (i.e., the peroxide).^30^ However, providing clues about solvent effects is not the main aim of this work, nor it appears to be a simple and straightforward task, given the complexity of the solvation processes that should be included.

A discussion on the effect of a second metal cation, which neutralizes the net charge of the anionic system, has been already attempted in ref (30). The presence of extra cations, leading to a peroxide-like M_2_O_2_ stoichiometry, is certainly expected to exert a strong stabilizing effect on the peroxide state. Nevertheless, isolated MO_2_^–^ species, although not long-lived in the electrochemical cell environment, might be formed before they can cluster and nucleate. The existence of a lower energy M^0^O_2_^–^ state could then alter not only the disproportionation pathway and the release of singlet O_2_ but also the mechanism of deposition and growth of the discharge products.

### 3.1 LiO_2_^–^ System

**Figure 2:**
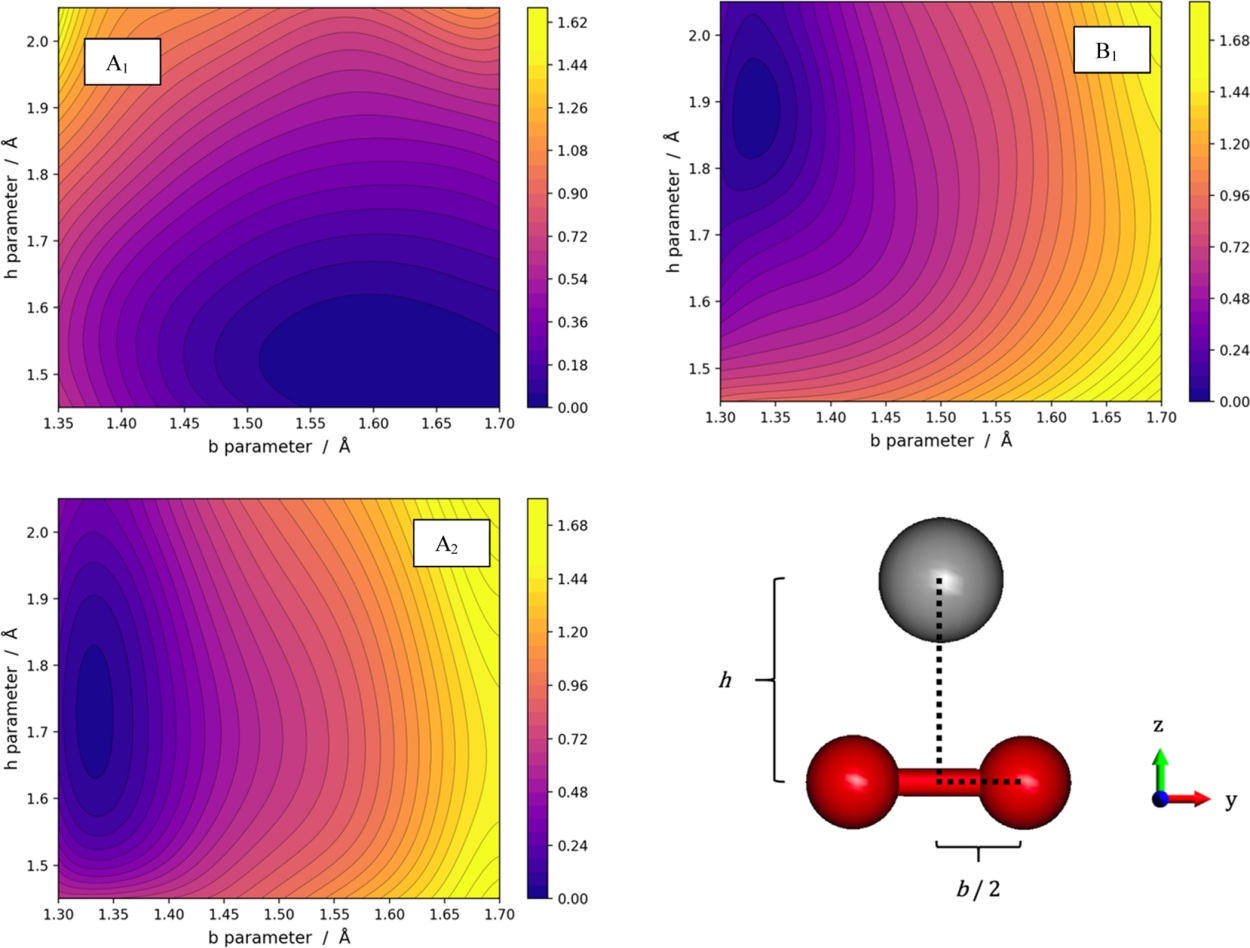
PESs of the three electronic states for the LiO_2_^–^ system. In each panel, the zero of the energies has been set at the minimum value. The energy scales are in electronvolt.

The PESs for the three electronic states of LiO_2_^–^ are illustrated in Figure 2. The peroxide A_1_ PES is in the top left panel, and it shows a deep and broad minimum, where the O–O distance (*b*) is large (see the structure reported in Table 1) and dominated by the O_2_^2–^·Li^+^ configuration. The other two panels show the PESs for the two superoxide states, where the minima are located at short O–O distances and the *h* values are between 1.7 and 1.9 Å depending on the state of symmetry. It is worth pointing out that while the peroxide (A_1_) minimum is broad and extends over a sizable range of O–O distances (∼0.15 Å), both superoxide minima (B_1_ and A_2_) occur only in a narrow range of O–O distances, practically only around 1.32–1.33 Å.

While the lowest-lying two superoxide states (A_2_) show certain flexibility in terms of Li–O distances with *h* ranging from 1.6 to 1.9 Å, the other (B_1_) becomes strongly repulsive when *h* becomes smaller than 1.7 Å. This is the consequence of the fact that the A_2_ state has one of its unpaired electrons occupying the π_x_^*^, which points toward the Li atom.

**Figure 3:**
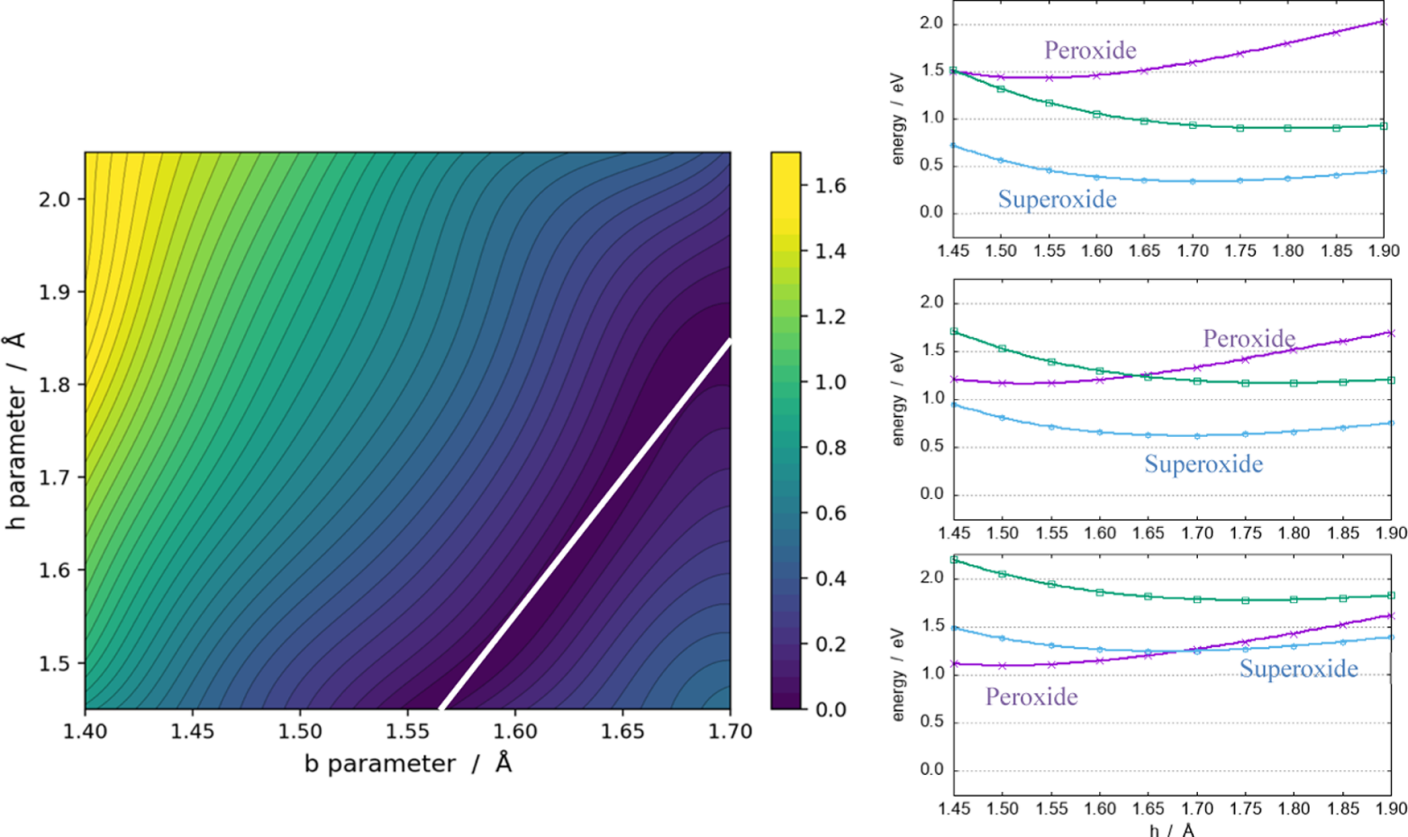
LiO_2_^–^ system. Left panel: |*E*(A_1_)–*E*(A_2_)| energy difference as a function of coordinates. Below the white line, the peroxide state is the ground one; above it, the superoxide state is the ground one. Right panels: vertical cuts of the PESs of Figure 2 as a function of *h* at different *b* values (from top to bottom: 1.4, 1.5, 1.65 Å). The colors are: violet, A_1_ (peroxide); green B_1_ (superoxide); cyan A_2_ (superoxide). The zero of the curves is set at the minimum value of the A_2_ surface.

The PESs reported in Figure 2 must intersect with each other and they are allowed to cross because the three states have different symmetries. The seam of the crossing points between the A_1_ and A_2_ states essentially divides the set of geometries into two regions: in one, the O–O distances are short and the superoxide state (A_2_) is the ground state. In the other region, the O–O distances are large, and the ground state is the peroxide (A_1_). The two regions are clearly identified in Figure 3 in the left panel, where we have reported the unsigned energy difference |*E*(A_1_)–*E*(A_2_)|. The white line is located approximately at the zero contour, thereby separating the region where the superoxide dominates (above the line on the left) and the one where the peroxide dominates (below the line on the right).

The evolution of the PESs of the electronic states along the geometric changes is described by the series of plots reported in the right panels of Figure 3, where we show three cuts through the electronic states that are taken at fixed *b* (i.e., vertically within the squares of Figures 2 and 3). At short values of *b* (1.4 Å, top panel), the three states do not cross. An increase in the O–O distance leads the A_1_ state (violet) to cross with the first superoxide (B_1_, cyan). Increasing the O–O distance further lowers the energy of the peroxide (A_2_, violet) so that it also crosses the low-lying superoxide (B_1_) state; for short values of the M–O distance, it becomes the ground state of the system.

As we mentioned before, small deviations from the *C*_2*v*_ symmetry do not change the three PESs in a significant way. Nevertheless, it is interesting to explore such deviation and its effect. When setting the angle between *h* and *b* to be slightly less than 90°, the symmetry of the system is lowered from *C*_2*v*_ to *C*_*s*_. Symmetry lowering leads the A_1_ and B_1_ electronic states to become A′ and the A_2_ one to become A″. This implies that, while the A_1_ state is still allowed to cross the A_2_ one, it cannot intersect the B_1_ one anymore because the same symmetry implies an avoided crossing. In other words, the crossing points that we have shown to occur in *C*_2*v*_ symmetry between the A_1_ and B_1_ states appear to live on the seam of a conical intersection.

**Figure 4:**
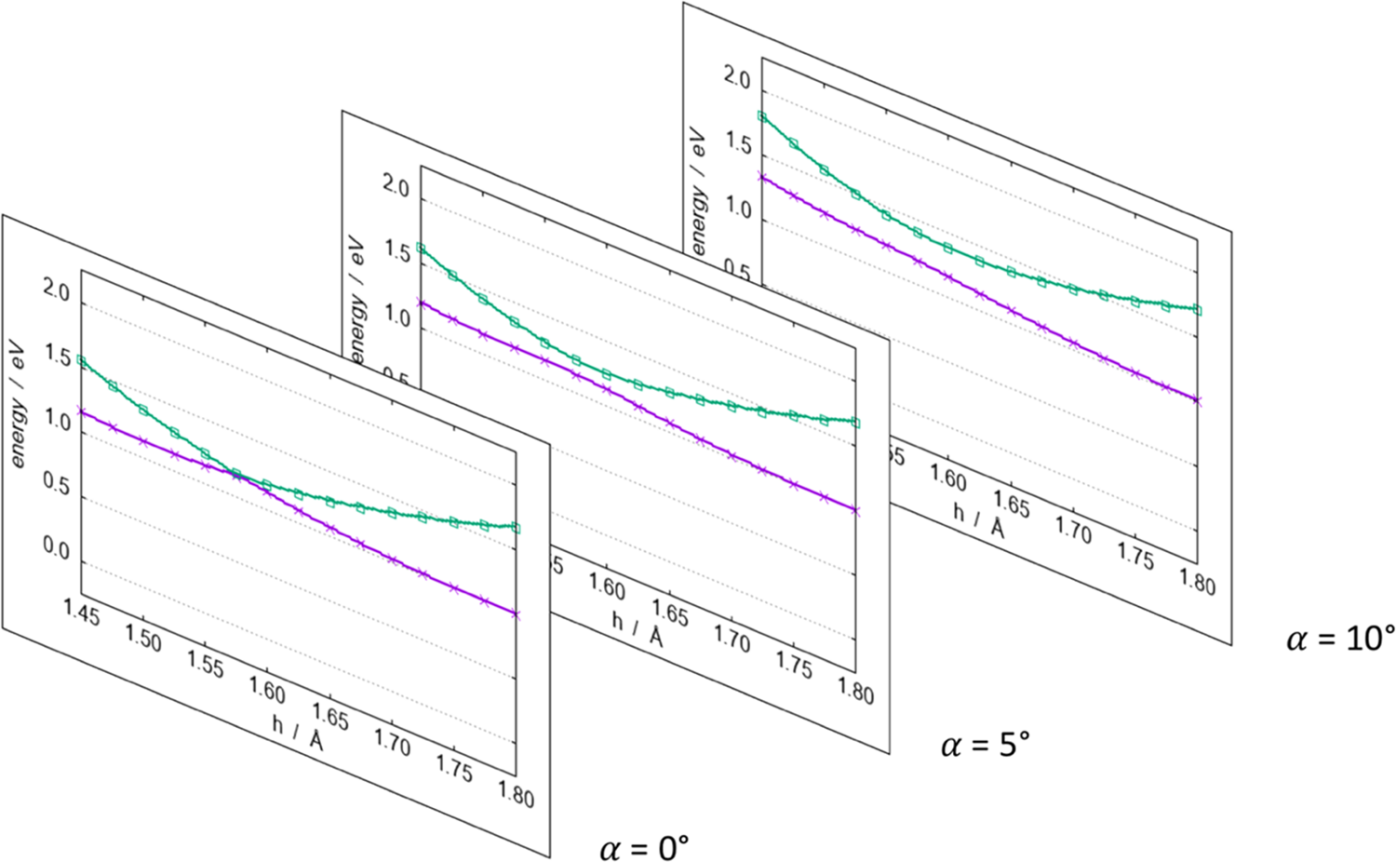
LiO_2_^–^ system. Vertical cuts of the PESs of Figure 2 as a function of *h* at *b* = 1.45 Å for different deviations from orthogonality. The colors are as follows: violet, ground state; green, excited state. The zero of the energies is set at the minimum value of the A_2_ surface.

As an example, we report in Figure 4 three different vertical cuts of the A_1_ and B_1_ PES for three values of the deviation from 90°. When the deviation is zero, the two surfaces intersect at *h* = 1.55 Å. As soon as the deviation increases, the two surfaces avoid each other because of the same symmetry (A′). An extended representation of the crossing seam of the A_1_ and B_1_ states in *C*_2*v*_ symmetry, in the same fashion, as shown in Figure 3, is reported in Section S6 of the SI.

### 3.2 NaO_2_^–^ System

The electronic states described for the Li system partially match the Na one. The two systems present an analogous set of electronic states, albeit the energy values and geometries obviously change.

**Figure 5:**
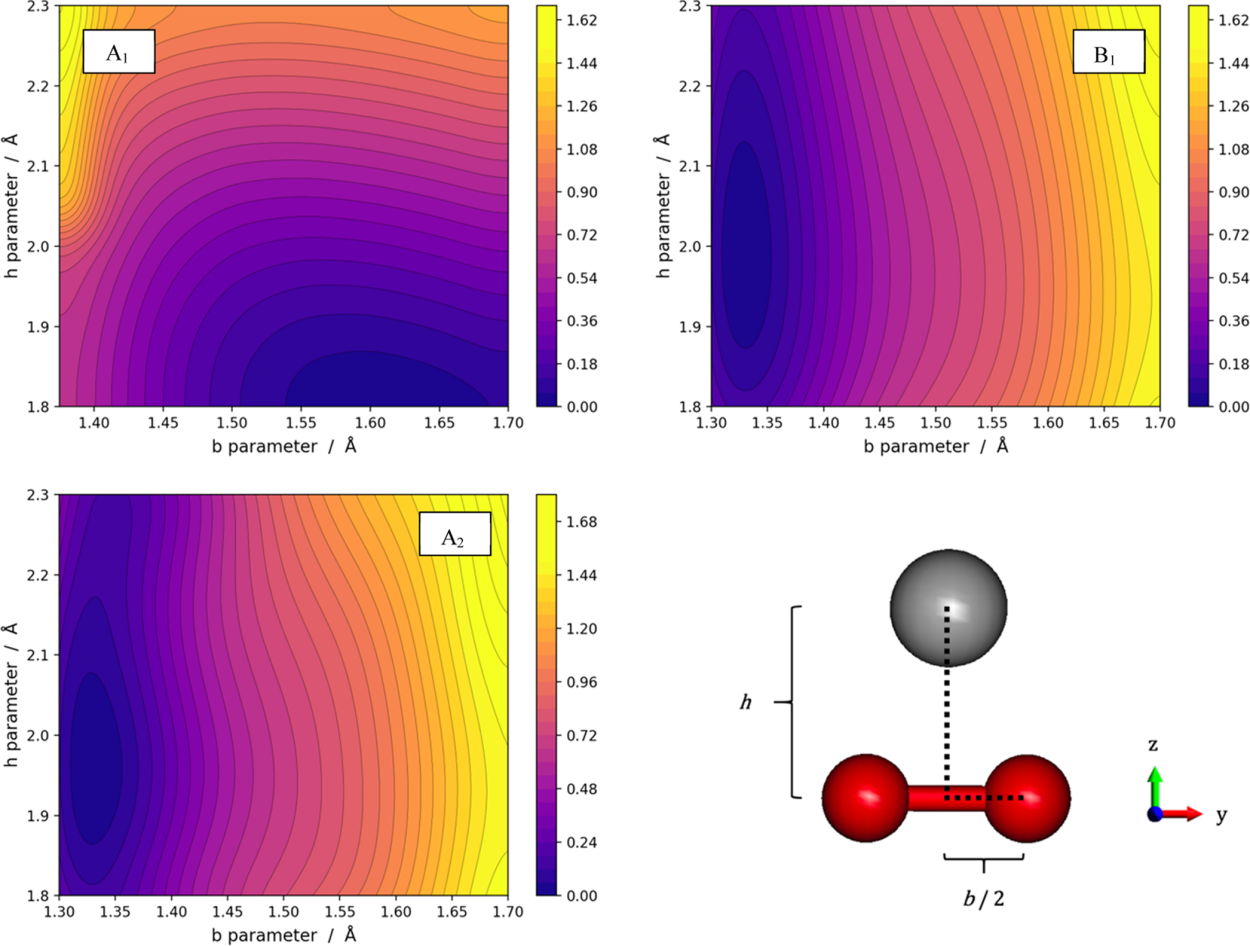
PESs of the three electronic states from the NaO_2_^–^ system. In each panel, the zero of the energies has been set at the minimum value. The energy scales are in electronvolt.

The three PESs for the NaO_2_^–^ system are presented in Figure 5. The top left panel illustrates the behavior of the *A*_1_ state (peroxide) with its broad and deep minimum at large O–O distances. The two superoxide states (B_1_ and A_2_) have their minima in a very narrow range of O–O distances centered around 1.32 Å. Such minima occur on a much wider range of M–O distances than in the case of Li. The NaO_2_^–^ complex in these two superoxide states is characterized by large flexibility of the Na position with *h* ranging from 1.9 to 2.3 Å. It is worth noting that, different from the Li case, the two superoxide states (A_2_ and B_1_) have very similar shapes. In other words, the two superoxide states appear to be substantially described by two parallel potential energy surfaces whose energetic difference is ∼0.3 eV.

In the NaO_2_^–^ system, the peroxide state (A_2_) is more destabilized in comparison with Li. The A_2_ and A_1_ states cross only when the O–O distance exceeds 1.7 Å.

**Figure 6:**
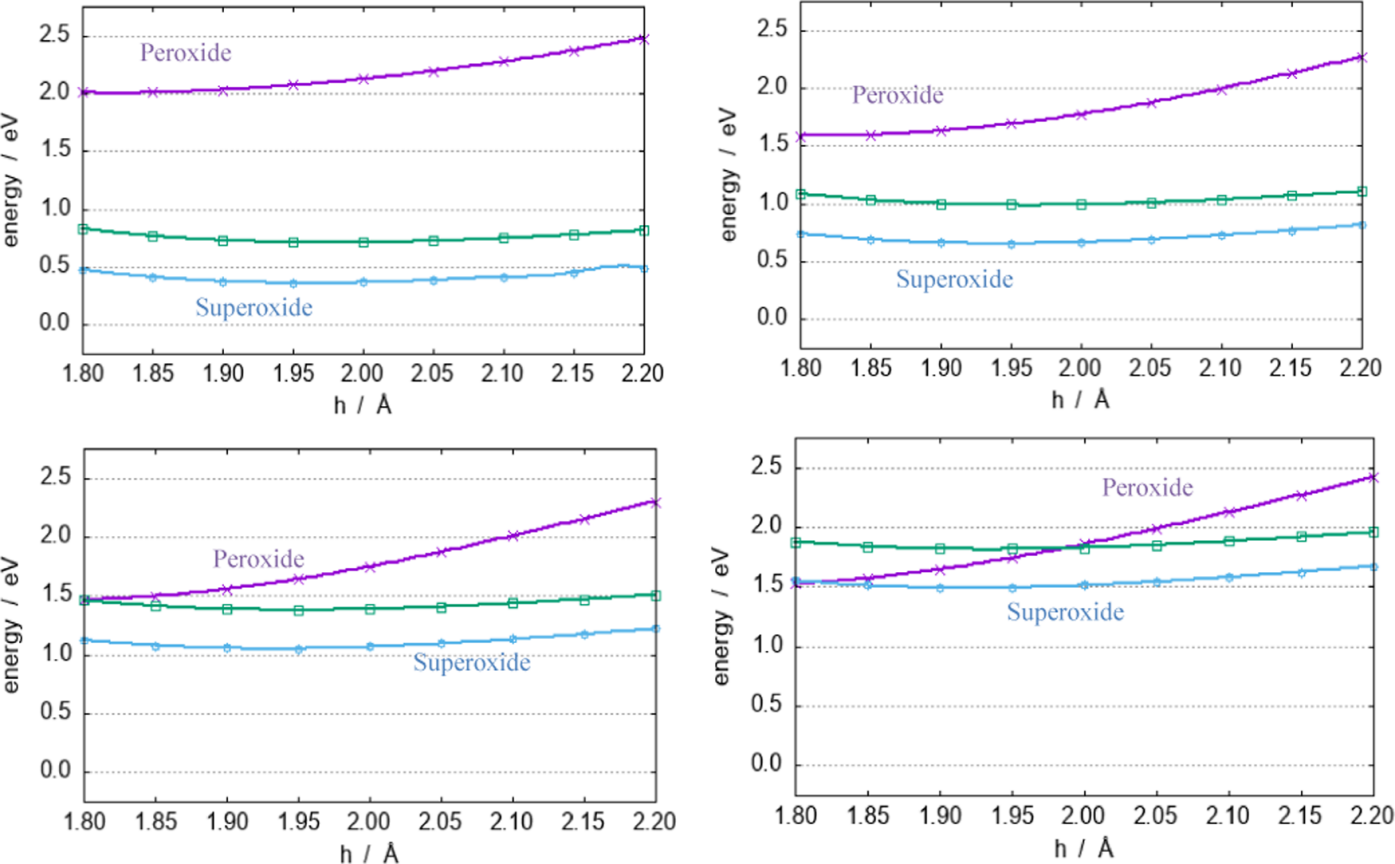
Vertical cuts of the PESs of Figure 4 as a function of *h* at different *b* values (in reading order: 1.4, 1.5, 1.6, and 1.7 Å). The colors are as follows: violet, A_1_ (peroxide); green B_1_ (superoxide); and cyan A_2_ (superoxide). The zero of the energies is set at the minimum of the A_2_ surface.

An overview of the dependence of the NaO_2_^–^ electronic states as a function of the O–O distance is provided in Figure 6 through the plots of the vertical cuts of the PESs of Figure 5. Increasing gradually, the O–O distance leads to a reduction in the energy of the A_1_ state (violet); between 1.6 and 1.7 Å, it crosses the B_1_ state, but even with O–O distances as large as 1.7 Å, the superoxide state is never the ground state. The tendency to favor the superoxide state seems to increase with increasing the size of the metal; hence, a decrease in its ionization potential, as noted in previous experiments.^37,38^

### 3.3 Comparison to Neutral (su)peroxide Systems

The tendency to favor the superoxide state when increasing the size of the alkali metal in the anionic MO_2_^–^ systems shows a clear analogy with the general, well-known trend of increased stability of neutral alkali superoxide vs peroxide phases.^9,26,37−39^ Interestingly, a somehow similar trend is observed in ref (27), where the dissociation of neutral MO_2_ superoxide (M = Li, Na, K) is investigated through multireference correlated ab initio methods. Here, along the OO–M dissociation coordinate, the energy curves relative to the formation of neutral M^0^ atoms are remarkably closer to the equilibrium ground state energy for Na than for Li. Also, the same study confirms the tendency of M^0^ states to reach a minimum at much larger OO–M distances than that of the M^+^ ones, a fact mainly ascribed to the different nature of the leading Coulombic/van der Waals interaction. Experimentally determined dissociation energies of alkali superoxides are in qualitative agreement with these findings.^21,40^

## 4 Concluding Remarks

In this work, we presented accurate gas-phase calculations on the LiO_2_^–^ and NaO_2_^–^ anionic trimers. These compounds are key ingredients of the chemistry of metal–air batteries. While from their charge state, they appear to be peroxides, they hide an unexpected complexity, and the true nature of their ground state is that of the superoxide. The complexity of the electronic structure of these compounds is linked to the multiconfigurational nature of their electronic states. A single-reference method would almost inevitably lead to the conclusion that the overall singlet multiplicity of the molecule is bound to a closed shell arrangement of the electrons, yielding the M^+^O_2_^2–^ configuration that is a peroxide. As we have proven using multiconfigurational methods, their ground electronic state is an open-shell singlet diradical corresponding to an M^0^(↑)O_2_^–^(↓) configuration that includes a superoxide anion.

By exploiting the inherent *C*_2*v*_ symmetry of the triatomic system, we have traced the behavior of the PESs of the first three electronic states as a function of the internal coordinate of the molecule. We have shown that the nature of the ground state depends on the O–O distance: at small values, the lowest-lying electronic state is the superoxide, and it becomes a peroxide only when the O–O distance exceeds a certain threshold, which is 1.6 and 1.7 Å for Li and Na, respectively. The change in the nature of the electronic ground state is due to the gradual lowering of the energy of the peroxide state while increasing the O–O distance. During this geometric change, the peroxide gradually decreases its energy and crosses both superoxide states.

The presence of a low-lying M^0^ superoxide channel and its energetic dominance for a large portion of the possible triatomic geometries of the MO_2_^–^ geometries is something that cannot be overlooked. The occurrence of this channel in the possible products of the superoxide disproportionation reaction might have important consequences for the understanding of the parasitic processes leading to cell death. In particular, the presence of this low-energy channel might lower the overpotentials needed to induce the formation of singlet oxygen by a significant amount, thereby explaining why this reactive species has been found in practice despite the unfavorable energetic of its formation from superoxides.

## Supporting Information Available

Additional specifications on the electronic wavefunctions and additional calculations for triplet spin multiplicity and larger active space (PDF)

## Notes

The data that support the findings of this study are available from the corresponding author upon reasonable request.
