# Supplementary material for: Study of the Electronic Structure of Alkali Peroxides and Their Role in the Chemistry of Metal–Oxygen Batteries
Source: J Phys Chem A. 2021 Oct 15;125(42):9368–76. doi: 10.1021/acs.jpca.1c07255 (PMC8558866; doi:10.1021/acs.jpca.1c07255)
Supplement: Supplementary file 1 — jp1c07255_si_001.pdf [file jp1c07255_si_001.pdf]

A Study of the Electronic Structure of Alkali Peroxides and Their Role in the Chemistry of Metal-Oxygen Batteries.

Adriano Pierini,[1] Sergio Brutti[1,2] and Enrico Bodo[1,]*

[1]Department of Chemistry, University of Rome "La Sapienza", P. A. Moro 5, 00185 Rome, Italy

[2]GISEL—Centro di Riferimento Nazionale per i Sistemi di Accumulo Elettrochimico di Energia, INSTM via G. Giusti 9, 50121 Firenze, Italy

*Corresponding author: enrico.bodo@uniroma1.it

Supporting information

S1 Active Orbitals in the C_{2v} symmetry

Symmetry of the 7 valence atomic orbitals to the Complete Active Space for each irreducible representation in the C_{2v} group:

a_1	b_1	b_2	a_2
O p_z ($\rightarrow \pi_z$)<br>O p_y ($\rightarrow \sigma$)<br>Li s	O p_z ($\rightarrow \pi_z^*$)<br>O p_y ($\rightarrow \sigma^*$)	O p_x ($\rightarrow \pi_x$)	O p_x ($\rightarrow \pi_x^*$)

S2 Representative LiO_2^- wavefunctions

The optimized CASSCF wavefunctions of the LiO_2^- lowest electronic states (at selected geometries) are represented in terms of the CSFs active orbitals occupancy and their relative weights:

- A_1 (b=1.60; h=1.50)

a_1			b_1		b_2	a_2	coeff.	weight
2	2	0	2	0	2	2	-0.9417	0.8868
2	0	0	2	2	2	2	0.2309	0.0533
1	2	1	2	0	2	2	0.1906	0.0363
2	1	1	2	0	2	2	-0.1210	0.0146

Leading configuration: $(\mathrm{O}\ \pi_z)^2(\mathrm{O}\ \pi_x)^2(\mathrm{O}\ \sigma)^2(\mathrm{O}\ \pi_z^*)^2(\mathrm{O}\ \pi_x^*)^2$ $(\mathrm{Li\ s})^0(\mathrm{O}\ \sigma^*)^0$

- A_2 (b=1.35; h=1.75)

a_1			b_1		b_2	a_2	coeff.	weight
2	2	1	2	0	2	1	0.9986	0.9973

Leading configuration: $(\mathrm{O}\ \pi_z)^2(\mathrm{O}\ \pi_x)^2(\mathrm{O}\ \sigma)^2(\mathrm{O}\ \pi_z^*)^2(\mathrm{O}\ \pi_x^*)^1$ $(\mathrm{Li\ s})^1(\mathrm{O}\ \sigma^*)^0$

- B_1 (b=1.35; h=1.85)

a_1			b_1		b_2	a_2	coeff.	weight
2	2	1	1	0	2	2	0.9997	0.9994

Leading configuration: $(\mathrm{O}\ \pi_z)^2(\mathrm{O}\ \pi_x)^2(\mathrm{O}\ \sigma)^2(\mathrm{O}\ \pi_z^*)^1(\mathrm{O}\ \pi_x^*)^2$ $(\mathrm{Li\ s})^1(\mathrm{O}\ \sigma^*)^0$

S3 Representative NaO_2^- wavefunctions

The optimized CASSCF wavefunctions of the NaO_2^- lowest electronic states (at selected geometries) are represented in terms of the CSFs active orbitals occupancy and their relative weights:

- A_1 (b=1.60; h=1.95)

a_1			b_1		b_2	a_2	coeff.	weight
2	2	0	2	0	2	2	-0.9556	0.9132
2	0	0	2	2	2	2	0.2339	0.0547
1	2	1	2	0	2	2	0.1521	0.0231

Leading configuration: $(O\ \pi_z)^2(O\ \pi_x)^2(O\ \sigma)^2(O\ \pi_z{*})^2(O\ \pi_x{*})^2\ (Na\ s)^0(O\ \sigma{*})^0$

- A_2 (b=1.35; h=2.20)

a_1			b_1		b_2	a_2	coeff.	weight
2	2	1	2	0	2	1	0.9978	0.9957

Leading configuration: $(O\ \pi_z)^2(O\ \pi_x)^2(O\ \sigma)^2(O\ \pi_z{*})^2(O\ \pi_x{*})^1\ (Li\ s)^1(O\ \sigma{*})^0$

- B_1 (b=1.35; h=2.25)

a_1			b_1		b_2	a_2	coeff.	weight
2	2	1	1	0	2	2	0.9978	0.9956

Leading configuration: $(O\ \pi_z)^2(O\ \pi_x)^2(O\ \sigma)^2(O\ \pi_z{*})^1(O\ \pi_x{*})^2\ (Li\ s)^1(O\ \sigma{*})^0$

S4 Higher spin multiplicities

For both the A_2 and B_1 singlet superoxide states, a corresponding triplet state is found to be very close in energy with respect to the open-shell singlet. Throughout the explored PES, the singlet-triplet splitting turned out to be within the range of 0.1 eV. In Figure S1 we report the energies of the singlet and triplet A_2 and B_1 PESs at the value b=1.30 Å.

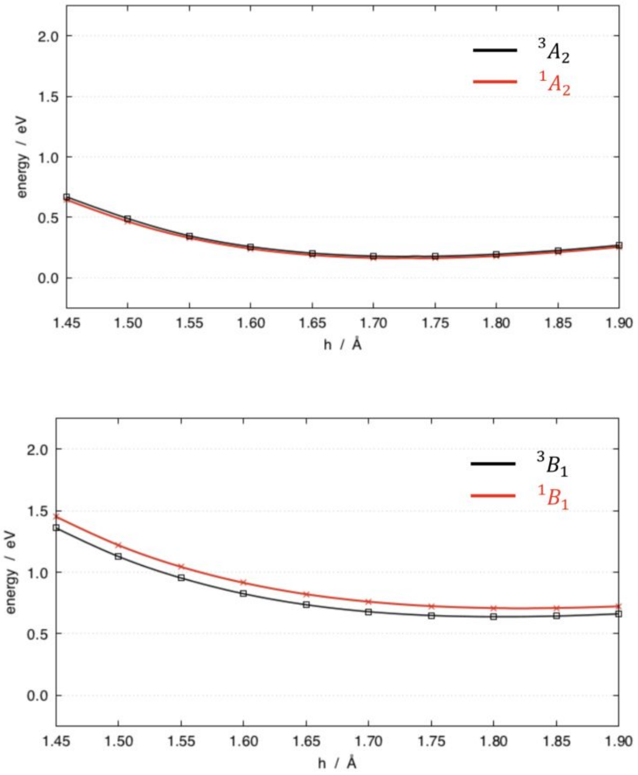

Figure S1: LiO_2^- system. PES cuts along *h* showing the singlet-triplet energy splitting in two different electronic states.

Since the reactants of (R4) can be an overall singlet or triplet, the same spin multiplicities can emerge in the products. This can be obtained either by combining a singlet O_2 with both singlet and triplet MO_2^-, or triplet O_2 with a singlet MO_2^-. Hence, the triplet MO_2^- product only

enters in the reactive pathway towards singlet oxygen, which is energetically disfavored by ca. 0.9 eV with respect to triplet oxygen.

S5 Test on the size of the active space

The choice of the active space is rather arbitrary. It should include all possible orbitals that give rise to significant occupations in the electronic states of interest for the chemical problem at hand. For our purposes, the choice of 7 active valence orbitals fulfils this obligation. However, the *2p* orbitals of Li are close in energy to the *2s* one and their inclusion is worth to be explored. We have repeated some of the calculations using a larger [10,10] active space that includes the *2p* orbitals of Li, to check whether their occupation gives rise to other electronic states which might be relevant for the reactions of superoxides. In Figure S3 we report three cuts of the resulting six lowest potential energy surfaces, at the same *b* values as in Figure 3 of the main text (b = 1.40 Å, 1.50 Å, 1.65 Å).

When we include the *2p* orbitals, three new electronic states appear at low energies, one for each of the a_1, b_1 and a_2 irreducible representation. They all correspond to superoxide states, with singly occupied p_y, p_x and p_z lithium orbitals. We will design them as $2A_1$, $2B_1$ and $2A_2$ and we have indicated them using dot-dashed lines in Figure S3.

The ground superoxide state $1A_2$ due to *2s* occupancy (cyan, full line), is more than 0.5 eV lower in energy than the $2A_2$ state due to the *2p* occupancy (cyan, dashed line) which is characterized by a PES that appears to be simply shifted upwards in energy with respect to the former. The same happens to the $2A_1$ state (violet, dashed line) that lies at higher energies, but has a PES that is almost parallel to the $1A_1$ one.

Interestingly, the two B_1 states arising from either the *2s* or *2p* occupancy (green) are close to each other, and they clearly interact in the range of h = 1.45 ÷ 1.65. However, the B_1 states represent electronically excited superoxide which we have included for sake of completeness but should play a minor role in the reactive behavior of (su)peroxides.

Overall, the chemistry of the low-lying electronic configurations are mainly dominated by the Li *2s* orbital, in agreement with the conclusions of ref. [27]. The little relevance of Li *2p* orbitals in the active space of neutral LiO_2 was also noticed in ref. [20].

As the low-lying superoxide state *1A₂*, seems unaffected by an expansion of the active space, the discussion in the main text on the nature of the ground state of LiO_2^- when varying the *b* and *h* parameters remains substantially unaltered and it is possible to limit the discussion to the electronic states arising from the *2s* occupancy.

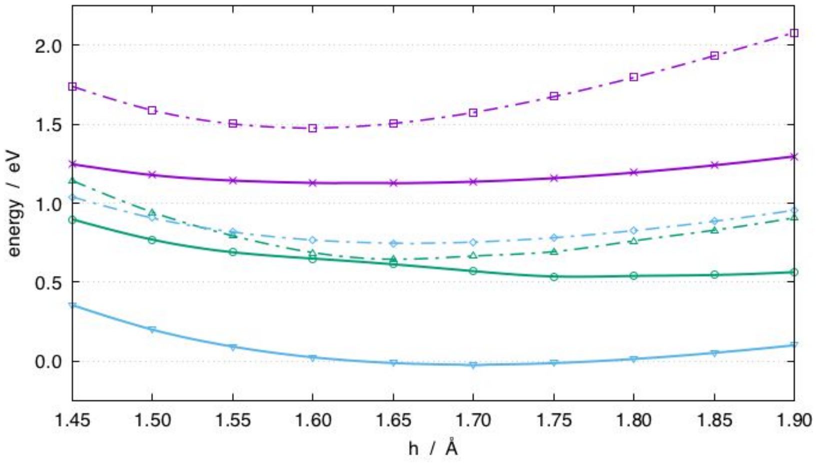

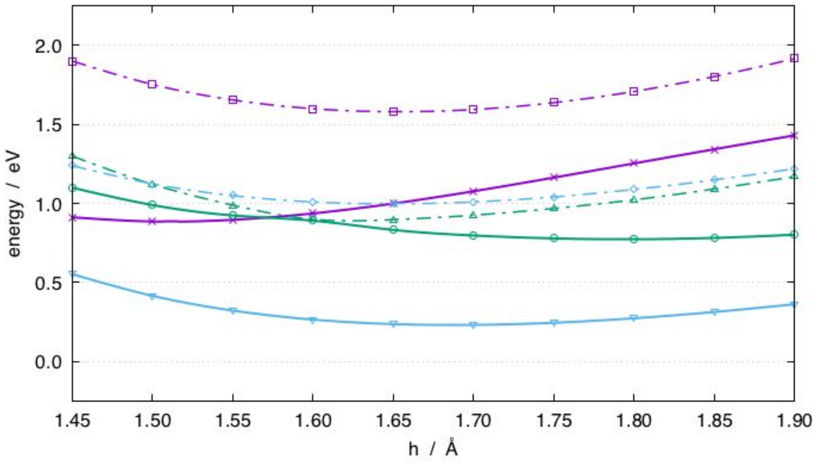

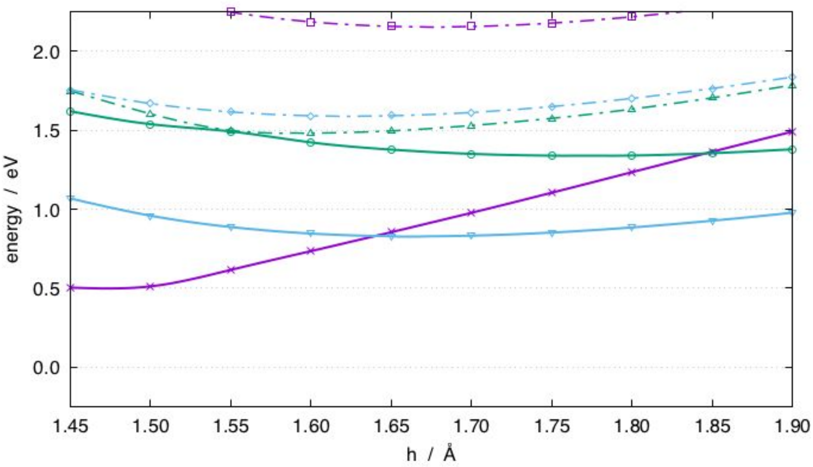

Figure S2: LiO_2^- system. PES cuts at b = 1.40 Å, 1.50 Å, 1.65 Å (from top to bottom) obtaine using a [10,10] active space. Solid lines: electronic states with *2s* occupancy. Dashed lines: electronic states with *2p* occupancy.

S6 Crossing of the A_1-B_1 states of LiO_2^-

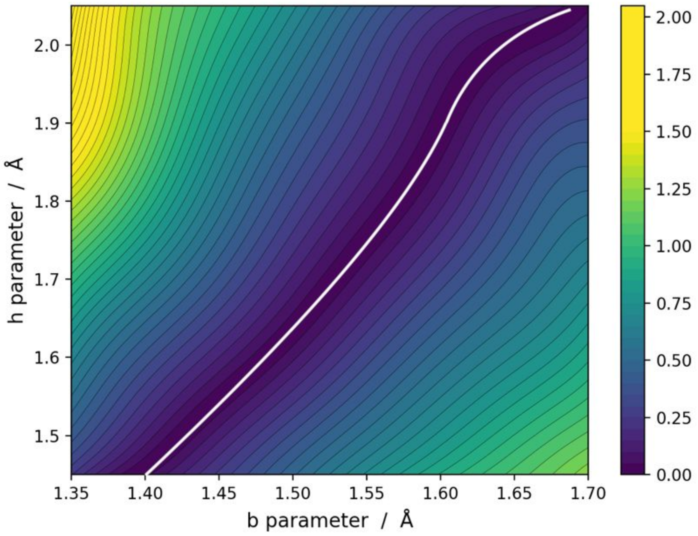

Figure S3: LiO_2^- system. $|E(A_1) - E(B_1)|$ energy difference as a function of coordinates. The white line is the crossing seam of the two states. Above the line $E(A_1) > E(B_1)$. The colormap scale is in eV.
